# Supplementary figures and images for: Root system architecture and environmental flux analysis in mature crops using 3D root mesocosms
Source: Front Plant Sci. 2022 Dec 15;13:1041404. doi: 10.3389/fpls.2022.1041404 (PMC9800027; doi:10.3389/fpls.2022.1041404)

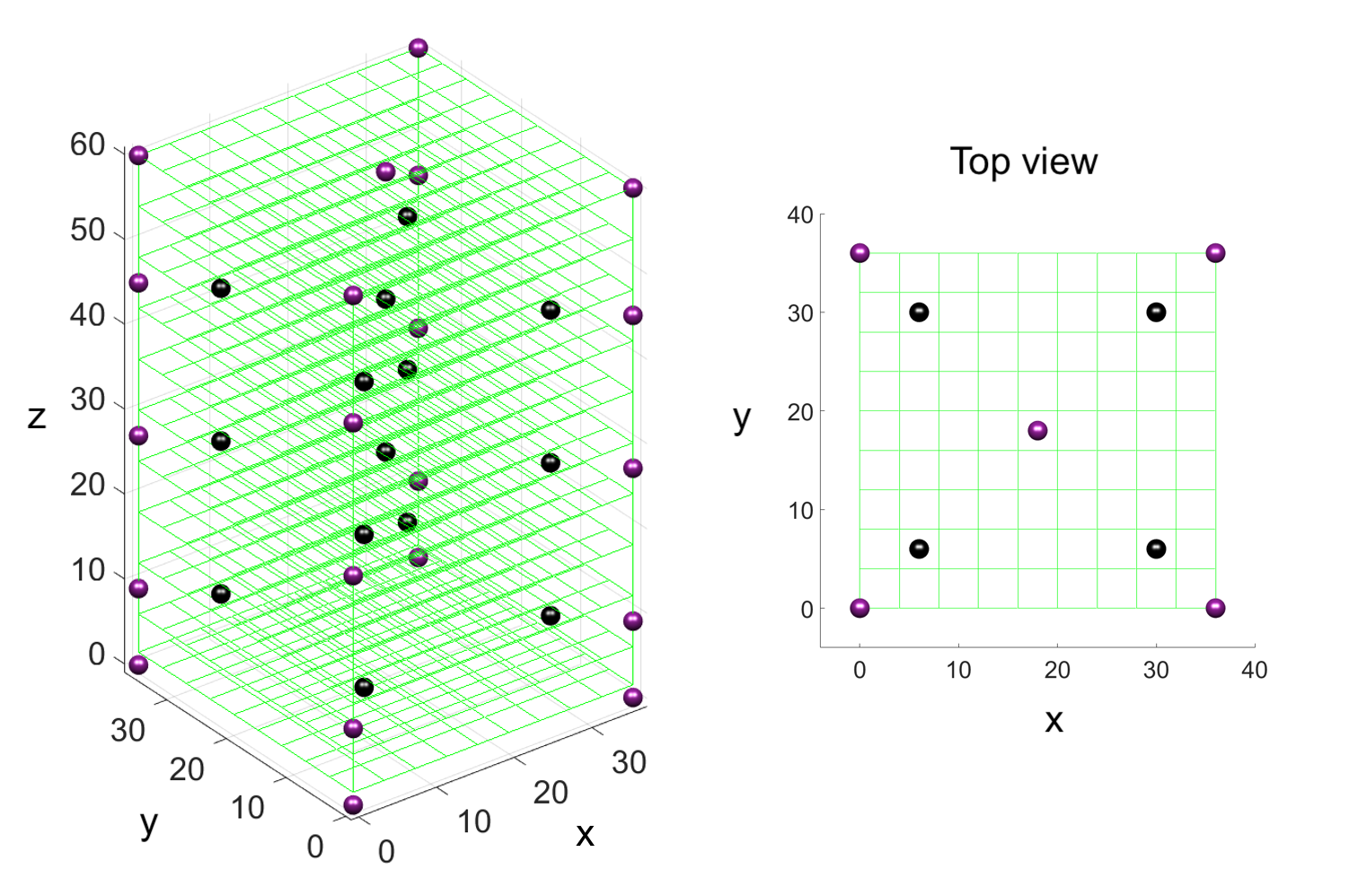

Supplement: Supplementary Figure 1 — Interpolation of 3-dimensional environmental sensor data. [file Image_1.tif]

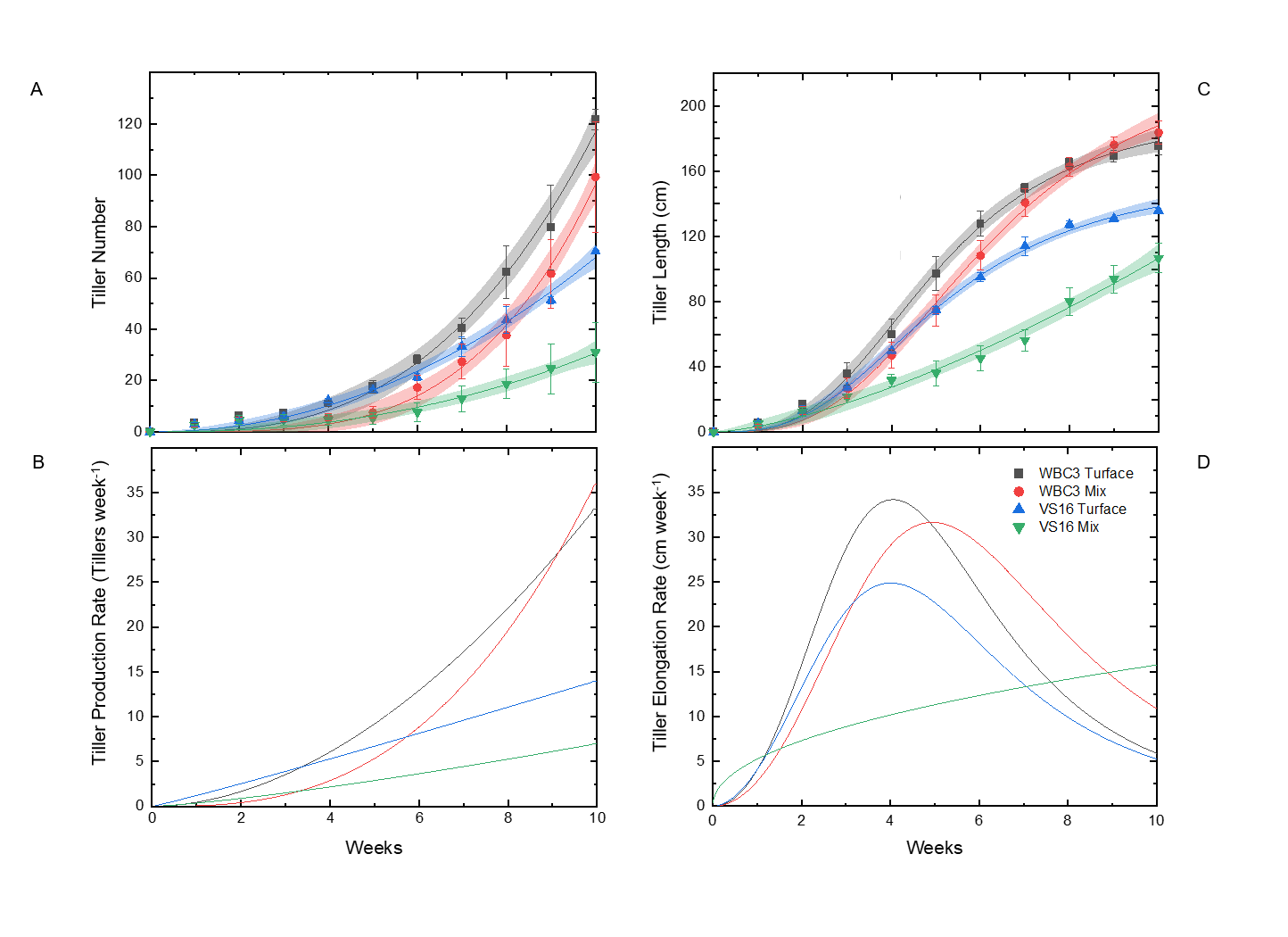

Supplement: Supplementary Figure 2 — Time course of shoot morphological responses of switchgrass in different growth media. [file Image_2.tif]

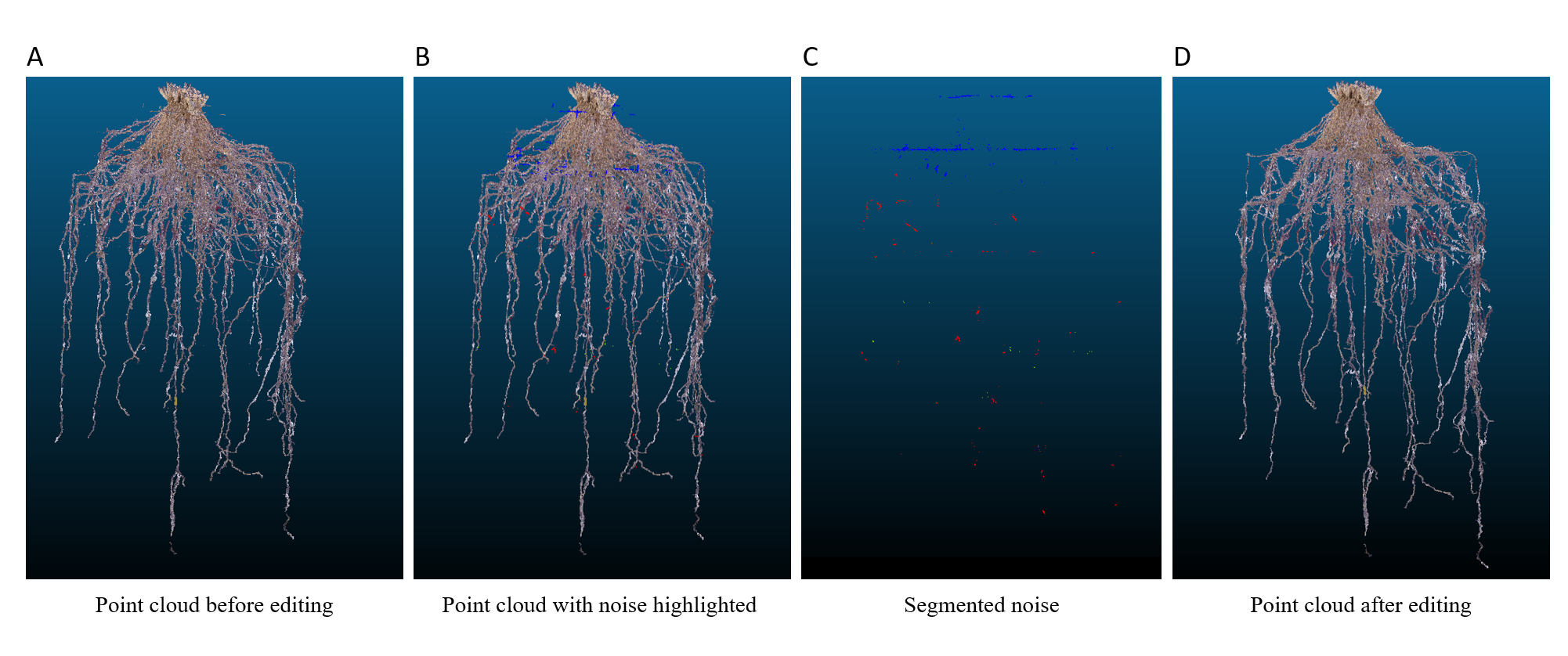

Supplement: Supplementary Figure 3 — Manual post-process cleaning of RSA point clouds. [file Image_3.tif]

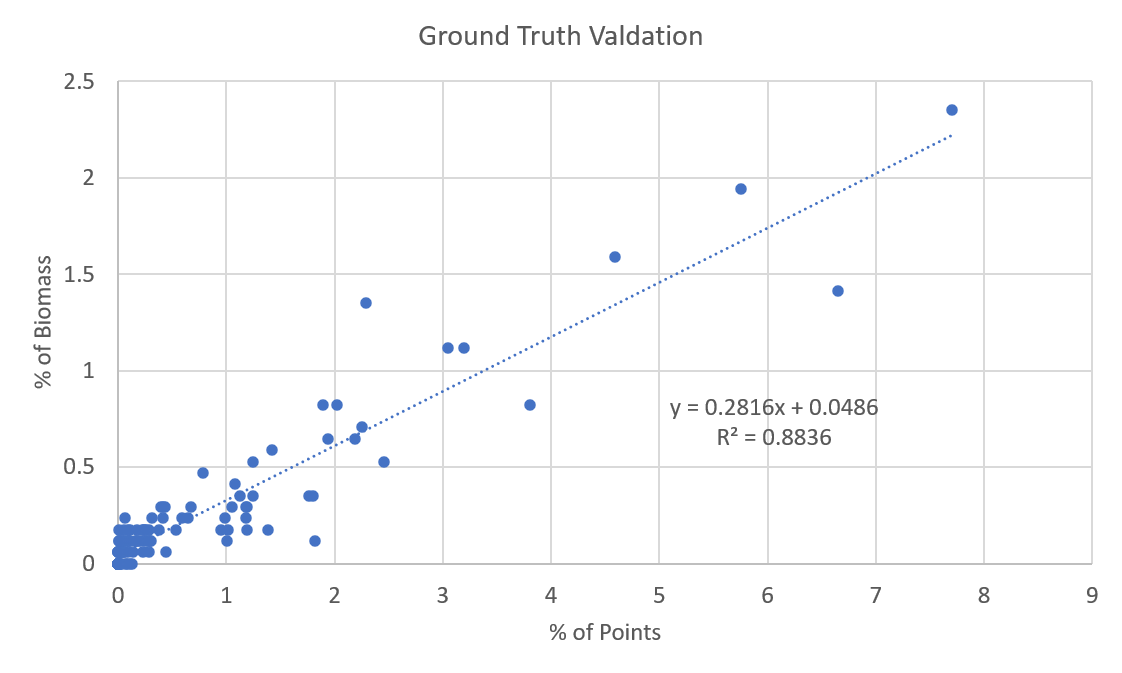

Supplement: Supplementary Figure 4 — Comparison of the biomass and point number located in each cuboid throughout the mesocosm growth zone. [file Image_4.tif]

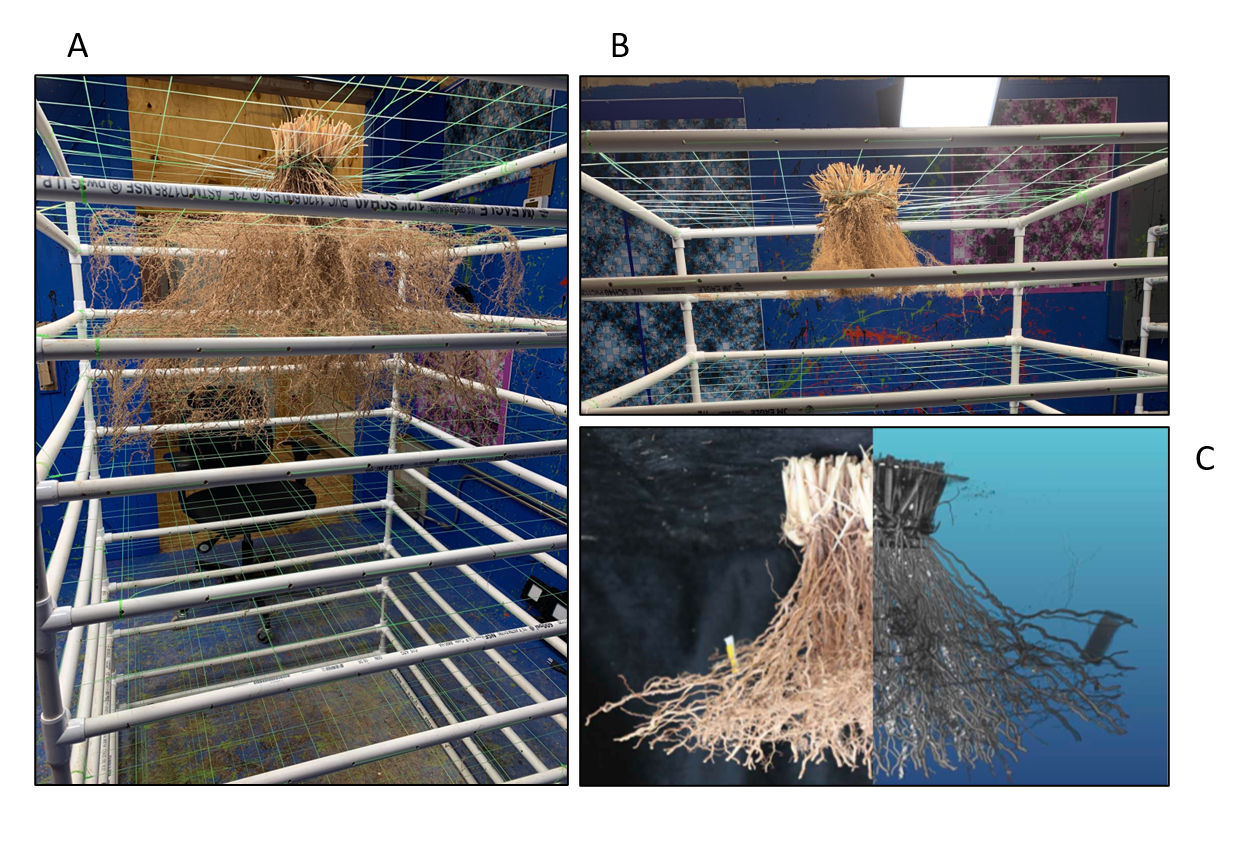

Supplement: Supplementary Figure 5 — Dissection of mesocosm grown root system for biomass measurements [file Image_5.tif]

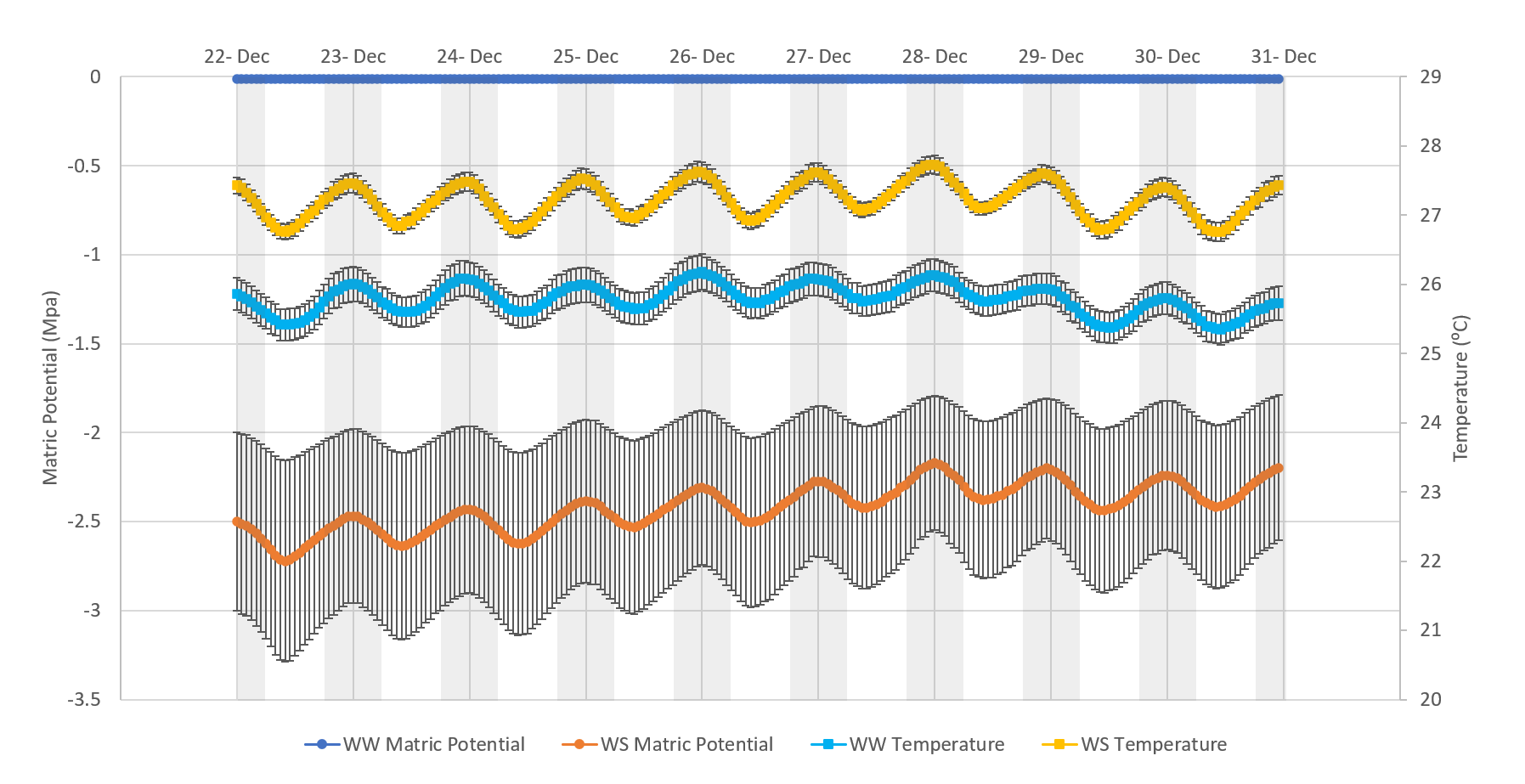

Supplement: Supplementary Figure 6 — Diurnal environmental fluxes in mesocosms across nine days. [file Image_6.tif]

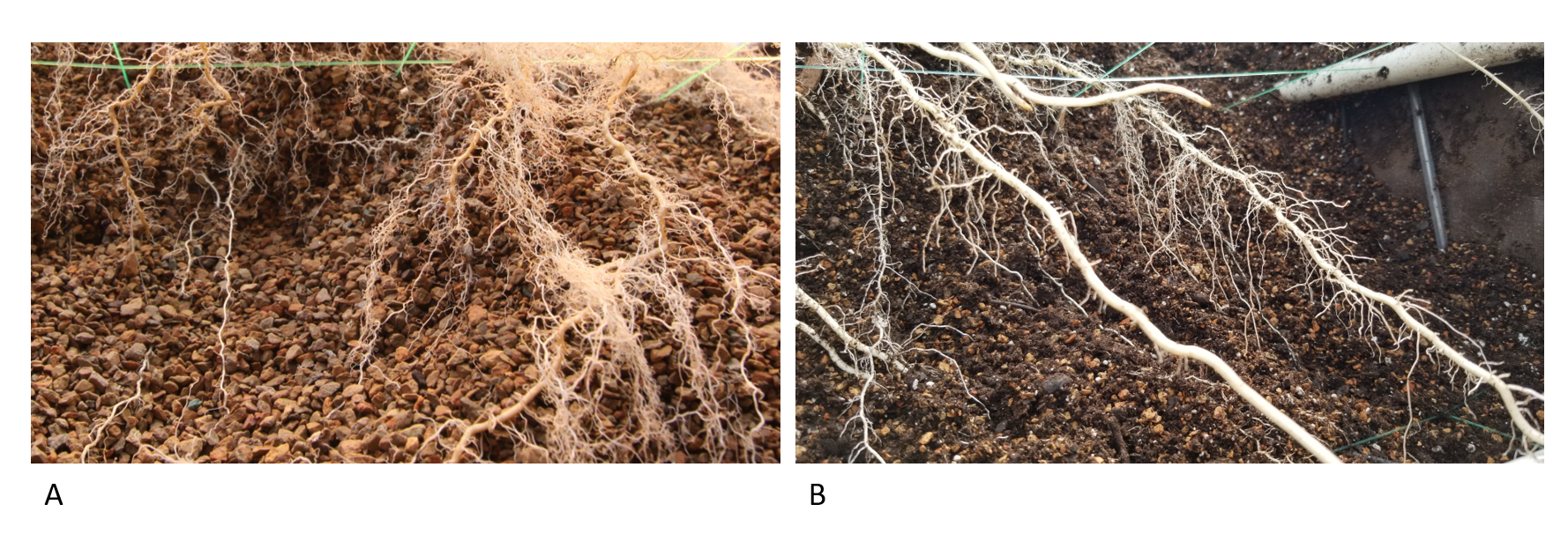

Supplement: Supplementary Figure 7 — Growth media effects on lateral root architecture [file Image_7.tif]

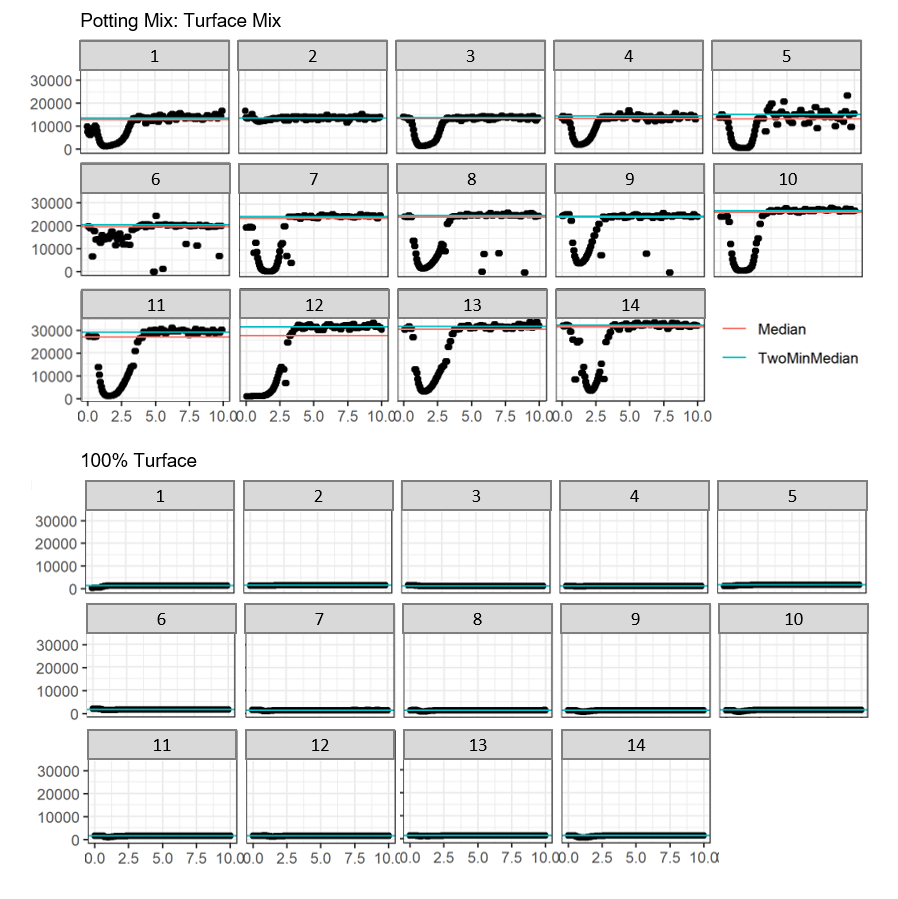

Supplement: Supplementary Figure 8 — Subterranean CO2 flux monitoring in mesocosms. [file Image_8.tif]
